# Direct Catalytic *N*‐Alkylation of α‐Amino Acid Esters and Amides Using Alcohols with High Retention of Stereochemistry

**DOI:** 10.1002/cssc.202100373

**Published:** 2021-05-07

**Authors:** Tao Yan, Ben L. Feringa, Katalin Barta

**Affiliations:** ^1^ Stratingh Institute for Chemistry University of Groningen Nijenborgh 4 9747 AG Groningen (The Netherlands; ^2^ Institute for Chemistry University of Graz Heinrichstrasse 28/II 8010 Graz Austria

**Keywords:** amino acid esters, borrowing hydrogen, chirality, *N*-alkylation, Ru catalysis

## Abstract

The direct functionalization of naturally abundant chiral scaffolds such as α‐amino acid esters or amides with widely abundant alcohols, without any racemization, is a demanding transformation that is of central importance for the synthesis of bio‐active compounds. Herein a robust and general method was developed for the direct *N*‐alkylation of α‐amino acid esters and amides with alcohols. This powerful ruthenium‐catalyzed methodology is atom‐economic, base‐free, and allowed for excellent retention of stereochemical integrity. The use of diphenylphosphate as additive was crucial for significantly enhancing reactivity and product selectivity. Notably, the only by‐product was water and both substrates could be potentially derived from renewable resources.

## Graphical abstract

***N*** **‐alkylation**: An efficient method for the base‐free *N*‐alkylation of amino acid esters and amides with easily accessible alcohols is presented. Ropivacaine is synthesized in high yield and excellent retention of the stereochemical integrity.

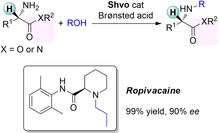

*N*‐alkyl amino acid esters and amides are frequently encountered chiral moieties in bioactive compounds (Figure 1). For example, Cilazapril^[1a]^ and Enalapril^[1b]^ are enzyme inhibitors, while Ximelagatran is an anticoagulant that has been investigated as an alternative for warfarin therapy.^[1c]^ Furthermore, Lidocaine and Bupivacaine are well‐known and widely used drug molecules that are included in the World Health Organization (WHO) list of essential medicines.^[2]^ Traditional pathways to obtain *N*‐alkyl amino acid esters commonly include reductive amination of aldehydes or nucleophilic substitution with alkyl halides (Scheme 1A).^[3]^ While the former pathway[^3b^, ^3c^] may be limited by the availability and stability of the aldehyde substrates as well as the formation of side products, the latter pathway[^3d^, ^3e^] generally suffers from poor selectivity and atom economy due to the formation of stoichiometric amount of undesired halogen‐containing salts.

**Figure 1:**
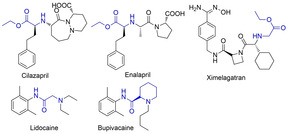
Important pharmaceutically active compounds containing a chiral *N*‐alkyl amino acid ester or amide moiety.

**Scheme 1:**
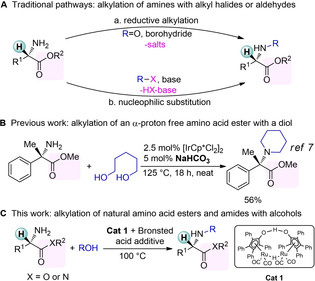
Synthesis of functionalized *N*‐alkyl amino esters and amides via *N*‐alkylation of amino acids and/or esters.

Alcohols are vastly abundant substrates^[4]^ and would be optimal alkylation agents and excellent alternatives to the commonly used alkyl‐halogenides or aldehydes. Despite tremendous developments in catalytic borrowing hydrogen strategies[^5^, ^6^] the direct *N*‐alkylation of natural amino acid esters via this methodology has remained largely unaddressed and there is lack of general methods that would allow for preserving the valuable chiral information in the amino acid backbone. While there are a number of examples that involve the use of chiral substrates,^[6f]^ to the best of our knowledge, only one example for the *N*‐alkylation of an unnatural amino acid ester has been reported employing an Ir‐based homogeneous catalytic system and 1,5‐pentane‐diol, which was limited to using NaHCO_3_ as base and a substrate that comprised a quaternary stereocenter (Scheme 1B).^[7]^ Indeed, most of the borrowing hydrogen methodologies rely on the use of a base for activation of the catalyst or substrates.^[6]^ Considering the use of natural amino acid esters as substrates, the chiral α‐carbon would be sensitive to racemization due to its acidic proton in the presence of a base, as previously described.^[8]^

In order to accomplish the challenging *N*‐alkylation of natural or synthetic amino acid esters with alcohols without racemization on α‐carbon, a robust, base‐free catalyst system is needed. Importantly such catalyst should be tolerant to strong chelating coordination of the highly functionalized amino acid ester or amide substrates or derived reaction intermediates (Scheme 1C) that may block important coordination sites and thus might slow or shut down catalysis.^[9]^

We have previously introduced the first homogeneous Fe‐catalyzed direct *N*‐alkylation of amines with alcohols (with Knölker's complex)^[10]^ without any addition of base.^[11]^ Next, we have also developed a powerful, base‐free method for the direct coupling of unprotected amino acids with alcohols^[12]^ as well as the direct amination of β‐hydroxyl acid esters^[13]^ and the decarboxylative *N*‐alkylation of cyclic amino acids with alcohols^[14]^ using the Ru‐based Shvo's catalyst^[15]^ (**Cat 1**). These findings serve as excellent starting point for establishing a novel catalytic methodology for the direct coupling of amino acid amides and esters with alcohols (Scheme 1). Initial study revealed that amino acid esters behave differently from unprotected amino acids, since the lack of free carboxylic acid moiety may influence the imine formation or reduction rates and transesterification may also occur at higher temperatures. The method requires unique reaction conditions (temperature, catalyst loading, potential additives) to allow for sufficiently facile imine formation and reduction without competing side reactions (i. e., transesterification, dialkylation) and avoiding racemization (Scheme 2).

**Scheme 2:**
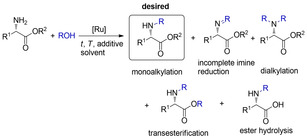
Reaction scheme of mono *N*‐alkylation of amino acid esters: desired product and possible side reactions.

To establish suitable conditions for the selective *N*‐alkylation of amino acid esters, we have selected phenylalanine pentyl ester (**1 a**) and 4‐methylbenzyl alcohol (**2 a**) as substrates and toluene as the solvent. The use of 0.5 mol% **Cat 1**, at 120 °C for 18 h, gave full conversion of **1 a**, but only 55 % selectivity of the desired mono‐alkylation product **3 a** (Table 1, entry 1) due to the formation of 35 % alkylated transesterification side product, as expected. With the benzyl ester of phenylalanine (**1 b**) the reaction was more sluggish, displaying only 69 % conversion (Table 1, entry 2). Lowering the reaction temperature to 90 °C with phenylalanine pentyl ester (**1 a**) gave minimal (<5 %) conversion (Table 1, entry 3), but no transesterification was seen.

**Table 1:** Direct *N*‐alkylation of l‐phenylalanine ester with 4‐methylbenzyl alcohol.^[a]^

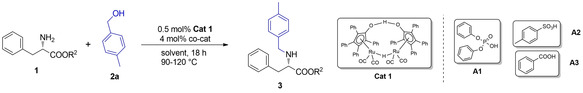								
Entry	R2/1	**2 a** [equiv.]	solvent	co‐cat.	T [°C]	Conv. 1 [%]	Sel. 3 [%]	
1	*n*‐pentyl/**1 a**	1.5	toluene	–	120	>99	**3 a**	55^[b]^
2	Bn/**1 b**	1.5	toluene	–	120	69	**3 b**	60
3^[c]^	*n*‐pentyl/**1 a**	2	toluene	–	90	<5	**3 a**	–
4^[c]^	*n*‐pentyl/**1 a**	2	toluene	**A1**	90	23	**3 a**	22
5^[c]^	*n*‐pentyl/**1 a**	2	toluene	**A2**	90	14	**3 a**	13
6^[c,d]^	*n*‐pentyl/**1 a**	2	toluene	**A3**	90	–	**3 a**	–
7	*n*‐pentyl/**1 a**	2	CPME	**A1**	100	26	**3 a**	23
8	*n*‐pentyl/**1 a**	2	THF	**A1**	100	18	**3 a**	10
9	*n*‐pentyl/**1 a**	2	heptane	**A1**	100	34	**3 a**	32
10	*n*‐pentyl/**1 a**	2	toluene	**A1**	100	63	**3 a**	62
11	*n*‐pentyl/**1 a**	4	toluene	**A1**	100	>99	**3 a**	93 (86), 96 % *ee*

[a] General reaction conditions: general procedure (see the Supporting Information, page S2), 0.5 mmol **1**, 1.5–4 equiv. **2 a**, 0.5 mol% **Cat 1**, 2 mL solvent, 18 h, 90–120 °C, isolated yield in parentheses, unless otherwise specified. Conversion and selectivity were determined by GC‐FID based on the integration ratio among amino acid contained moieties. [b] 35 % Alkylated transesterification side‐product was observed. [c] 24 h. [d] Decomposition of ester **1 a** was observed.

Realizing that at lower temperatures the competing transesterification step is much less pronounced, we have searched for alternative ways to enhance reactivity at 90 °C, by employing an appropriate Brønsted acid co‐catalyst. Previous studies have described cooperative catalysis in ruthenium‐catalyzed imine hydrogenation using chiral or achiral Brønsted acids,^[16]^ and we have earlier reported the enhancement of both the imine formation as well as the imine reduction step involved in the hydrogen borrowing sequence during the amination of β‐hydroxy acids employing the same Ru catalyst.^[13]^ Indeed, both the conversion of **1 a** (23 %) and **3 a** selectivity (22 %) improved upon addition of 4 mol% of diphenyl phosphate (**A1**) (Table 1, entry 4). The use of other Brønsted acids [*p*‐toluenesulfonic acid (**A2**) and benzoic acid (**A3**)] resulted only in moderate improvement (Table 1, entries 5 and 6) and screening a range of solvents at 100 °C with **A1** also showed poor results (Table 1, entries 7–9). Therefore, further optimization of reaction temperature and **2 a** amount in toluene was carried out, while keeping **A1** as the co‐catalyst in toluene (Table 1, entries 10 and 11). The best result was obtained at 100 °C, leading to full conversion of **1 a**, and 93 % selectivity (86 % isolated yield) of the desired product **3 a** with and 96 % retention of enantiomeric excess (*ee*; Table 1, entry 11). The positive results with diphenyl phosphate (**A1**) are likely due to the enhancement of imine formation^[17]^ as well as imine reduction^[16]^ steps involved in the hydrogen borrowing cycle.^[13]^

Next, the reaction scope was explored with selected examples of methyl‐, ethyl‐, isopropyl‐, and pentyl esters of diverse amino acids including phenylalanine (**Phe**), alanine (**Ala**), valine (**Val**), leucine (**Leu**), proline (**Pro**) and glutamic acid (**Glu**) (Scheme 3, details in the Supporting Information Table S2). Various esters of phenylalanine (**Ph**) were efficiently *N*‐alkylated with substituted benzyl alcohols (**2 a**, **2 b**, **2 d**) or pentanol (**2 c**) with excellent selectivity, high isolated yields, and high retention of *ee* in the corresponding products **3 c**–**3 f** (Table S2, entries 1–4). The functionalization of the alanine (**Ala**) backbone was evaluated using isopropyl and pentyl esters (Table S2, entries 6–9). Gratifyingly, the pentyl esters resulted in the formation of the corresponding mono‐alkylated products (**3 i**–**3 k**) in 66–79 % isolated yields and 92–97 % *ee* retention. Interestingly, slight racemization was observed for the isopropyl ester **3 h** (83 % *ee*). Next, the pentyl ester of valine (**Val**) was subjected to this optimized *N*‐alkylation protocol with benzyl alcohols (**2 a**, **2 b, 2 e**) affording the corresponding *N*‐alkyl analogues (**3 m**–**3 o**) with 82–87 % isolated yield and outstanding *ee* (99 %), while also the pentyl analogue **3 l** was obtained in 86 % isolated yield (Table S2, entries 10–13). Similarly, the ethyl ester of leucine (**Leu**) provided the desired products **3 p** and **3 q** with 96 and 94 % *ee*, respectively (Table S2, entries 14 and 15).

**Scheme 3:**
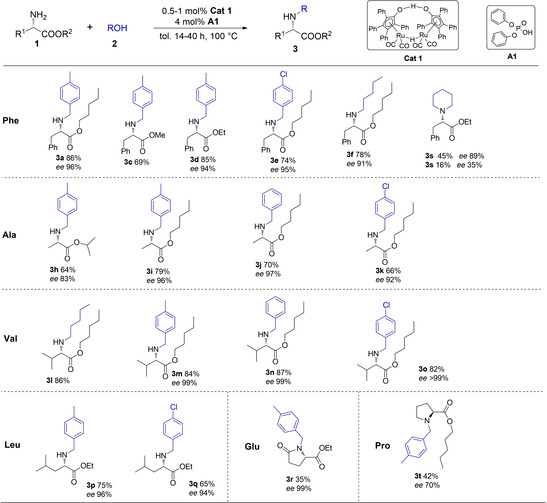
Direct *N*‐alkylation of l‐amino acid ester with alcohols.

Interestingly, the reaction of 4‐methyl‐benzyl alcohol (**2 a**) with glutamic acid (**Glu**) diethyl ester (**1 i**) lead to the formation of the cyclic 2‐pyrrolidinone derivative **3 r** with excellent stereoselectivity (99 % *ee*) albeit moderate isolated yield (35 %), due to an intramolecular amide formation (Table S2, entry 16). Finally, when the pentyl ester of proline (**Pro**) was selected to react with alcohol **2 a**, the corresponding product **3 t** was obtained with 42 % isolated yield and 70 % retention of *ee* (Table S3, entry 1).

We also performed a specific comparison between our Ru‐based catalytic system with the previously described Ir‐based system shown on Scheme 1B (Table S2, entry 17 vs. entry 18), in the direct alkylation of phenylalanine ethyl ester (**1 d**) with 1,5‐pentanediol (**2 f**). While our base‐free system (comprising **Cat 1** and **A1**) resulted in **3 s** in 48 % yield and 89 % *ee*, the base‐containing Ir system displayed lower conversion and significant racemization (16 % **3 s** yield, 35 % *ee)*.

Next, we addressed challenging examples of direct alkylation of amino acid amides with alcohols (Scheme 4). Gratifyingly, when prolinamide (**1 k**) and 4‐methylbenzyl alcohol (**2 a**) were selected as substrates (Scheme 4A), 83 % isolated yield of the desired *N*‐alkylation product *N*‐(4‐methyl)‐benzyl prolinamide (**3 u**) was obtained, with as low as 0.5 mol% **Cat 1** and 4 mol% **A1** (Table S3, entry 5). Unfortunately, only 59 % *ee* was obtained in this case, which indicates easier racemization of amino amides than esters in the working condition. Interestingly, when the Fe‐based **Cat 2** (Knölker's complex),^[10]^ previously applied in *N*‐alkylation by our group,^[11]^ was used in the same reaction, the cyclic product 4‐imidazonone **3 w** was obtained instead, via intramolecular nucleophilic attack of the iminium intermediate by the amide group (Scheme 4A), likely due to the sluggish iminium reduction step. This underscores the efficiency of our Ru‐based, base free method and the advantages of using the acid co‐catalyst **A1** expected to facilitate the important reduction step.

**Scheme 4:**
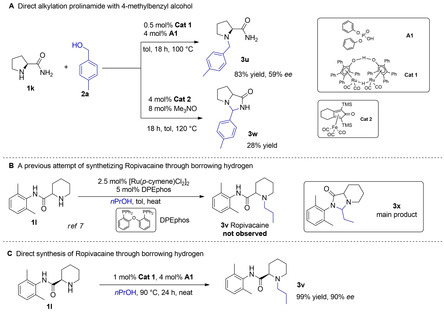
Comparison of product outcomes in the direct *N*‐alkylation of amino amides with alcohols.

Finally, to demonstrate the versatility of our methodology, we turned our attention to the straightforward synthesis of the pharmaceutical compound Ropivacaine (**3 v**).^[18]^ Previously, Leonard et al.^[7]^ attempted the direct Ru‐catalyzed *N*‐alkylation of the specific amino amide **1 l** with *n*‐propanol to access Ropivacaine (**3 v**); however, the imidazolidinone derivative (**3 x**) was obtained instead, similarly to what we observed with **Cat 2** in the case of **3 w** (Scheme 4A). Gratifyingly, with our catalytic system, quantitative yield of Ropivacaine (**3 v**) was obtained with 90 % *ee* when the *N*‐alkylation of amino‐amide **1 l** was performed in neat *n*‐propanol at 90 °C, using 1 mol% **Cat 1** and 4 mol% **A1** (Scheme 4C; Table S3, entry 6). This demonstrates the superior performance of our newly developed Ru‐catalyzed method for preparing pharmaceutically relevant *N*‐alkyl amino amides.

In summary, this study demonstrates an efficient method (0.5–1 mol% Ru loading) for the direct selective mono‐*N*‐alkylation of natural amino acid esters and amides with alcohols, with good to excellent yields and retention of the stereochemical integrity. This provides an excellent opportunity for the direct coupling of naturally abundant amino acid derivatives with widely accessible and potentially bio‐based alcohols. Moreover, this method allowed for the challenging direct *N*‐alkylation of amino‐acid amides, while taking advantage of the facile iminium reduction step, thus overcoming a common cyclization side reaction. The successful synthesis of Ropivacaine, which was prepared in quantitative yield and excellent *ee* retention, underscores the power of this methodology.

## Conflict of interest

The authors declare no conflict of interest.
